# Anxiety, depression, and quality of life of caregivers of palliative care patients with cancer

**DOI:** 10.15649/cuidarte.3670

**Published:** 2025-02-25

**Authors:** Alejandra María Alvarado García, Lina María Vargas-Escobar, Mauricio Arias-Rojas, Carlos Javier Avendaño-Vásquez, Cesar Antonio Consuegra-Pareja

**Affiliations:** 1 Profesor Asociado Universidad de Antioquia, Medellín Colombia. Universidad Antonio Nariño, Bogotá, Colombia. alejandra.alvarado@udea.edu.co alalvarado39@uan.edu.co Universidad de Antioquia Universidad de Antioquia Medellín Colombia alejandra.alvarado@udea.edu.co; 2 Profesor Titular Universidad El Bosque, Bogotá, Colombia. lmvargase@unbosque.edu.co Universidad El Bosque Universidad El Bosque Bogotá Colombia lmvargase@unbosque.edu.co; 3 Profesor Asociado Universidad de Antioquia. Medellín. Colombia. emauricio.arias@udea.edu.co Universidad de Antioquia Universidad de Antioquia Medellín Colombia emauricio.arias@udea.edu.co; 4 Profesor Asistente Universidad Antonio Nariño, Bogotá, Colombia. javierunvasquez76@gmail.com Universidad Antonio Nariño Universidad Antonio Nariño Bogotá Colombia javierunvasquez76@gmail.com; 5 Médico, Director de programa de Cuidado Paliativo, Cuidarte tu salud. Bogotá, Colombia. cuidadospaliativos@cuidartetusalud.com Programa de Cuidado Paliativo, Cuidarte tu salud Programa de Cuidado Paliativo, Cuidarte tu salud Bogotá Colombia cuidadospaliativos@cuidartetusalud.com

**Keywords:** Quality of life, Anxiety, Depression, Palliative Care, Caregivers, Calidad de vida, Ansiedad, Depresión, Cuidados Paliativos, Cuidadores, Qualidade de Vida, Ansiedade, Depressão, Cuidados Paliativos, Cuidadores

## Abstract

**Introduction:**

Palliative care has recently gained importance in the context of life-threatening diseases, such as cancer, that affect the mental health of patients and their caregivers. Psychological symptoms, such as anxiety and depression, are the most prevalent in this population.

**Objective:**

To determine the association between anxiety, depression, and quality of life of caregivers of palliative care patients with cancer.

**Materials and Methods:**

A quantitative, descriptive, and correlational design was used. A total of 190 primary caregivers recruited from Colombian hospitals and home care programs participated. The Quality of Life in Life-Threatening Illness-Family Carer (QOLLTI-FT) questionnaire, the Beck Depression Inventory-II (BDI-II), and the Beck Anxiety Inventory (BAI) were used. The analysis was performed with SPSS Statistics 26.0, using descriptive and inferential statistics.

**Results:**

The predominant sex was female, and the level of education was high school. The mean age of the caregivers was 44.15 years, and the mean age of the patients was 64.51 years. The average time as a caregiver was 33.79 ± 64.77 months. The results show correlations between anxiety and caregiver status (p≤0.000), anxiety and Information and Communication Technology (ICT) use (p≤0.006). There were also correlations between anxiety, quality of life, and caregiver status (p≤0.000) and between depression and total quality of life (p≤0.001).

**Discussion:**

Correlations were also found between depression and quality of life and between hours of daily care and the level of dependency of the cancer patient. This entails the need to manage the psychological symptoms as soon as the family member is diagnosed to prevent alterations in their quality of life that could affect their well-being.

**Conclusion:**

Caregivers with moderate or severe depression were more likely to report symptoms of sadness, punishment feelings, self-dislike, suicidal thoughts or wishes, indecisiveness, irritability, changes in appetite, concentration difficulty, and tiredness or fatigue. Among caregivers with mild depression, loss of interest in sex, agitation, and past failure were identified. Strategies for psycho-emotional counseling, education, and support for caregivers are needed.

## Resumen

**Introducción:**

El cuidado paliativo ha cobrado importancia recientemente en el contexto de las enfermedades como el cáncer, que amenazan la vida y afectan la salud mental de los pacientes y de sus cuidadores. Los síntomas psicológicos, como la ansiedad y la depresión, son los más prevalentes en esta población.

**Objetivo:**

El estudio tuvo como objetivo determinar la asociación entre la ansiedad, la depresión y la calidad de vida de cuidadores de pacientes con cáncer en cuidados paliativos.

**Materiales y métodos:**

Se utilizó un diseño cuantitativo, descriptivo y correlacional. Participaron 190 cuidadores primarios reclutados en hospitales y programas de atención domiciliaria en Colombia. Se utilizó el cuestionario Quality of Life in Life-Threatening Illness-Family Carer (QOLLTI-FT), el Beck Depression Inventory-II (BDI-II) y el Beck Anxiety Inventory (BAI). El análisis se realizó con SPSS Statistics 26.0, utilizando estadística descriptiva e inferencial.

**Resultados:**

El sexo femenino fue predominante y el nivel de escolaridad, secundaria. La edad media de los cuidadores fue 44.15 años y la de los pacientes, 64,51. El promedio de tiempo como cuidador fue de 33,79 ± 64,77 meses. Los resultados muestran correlaciones entre ansiedad y estado del cuidador (p≤0.000), ansiedad y uso de TIC (p≤0.006). También hubo correlación entre ansiedad, calidad de vida y estado del cuidador (p=0.00) y entre depresión y calidad de vida total (p≤0.001).

**Discusión:**

Se encontraron correlaciones entre la depresión y la calidad de vida y las horas de cuidado diario y el nivel de dependencia del paciente con cáncer. Esto implica manejar los síntomas psicológicos desde el diagnóstico del familiar para evitar alteraciones en su calidad de vida que afecten su bienestar.

**Conclusiones:**

En los cuidadores con depresión moderada y severa se presentó mayor frecuencia de síntomas de tristeza, sentimientos de castigo, disconformidad con uno mismo, pensamientos o deseos suicidas, indecisión, irritabilidad, cambios en el apetito, dificultad para concentrarse y cansancio o fatiga. En los cuidadores con depresión leve se identificó perdida de interés en el sexo, agitación y fracaso. Se necesitan estrategias de asesoramiento psicoemocional, educación y apoyo a los cuidadores.

## Resumo

**Introdução:**

Os cuidados paliativos ganharam importância recentemente no contexto de doenças fatais, como o câncer, que afetam a saúde mental dos pacientes e seus cuidadores. Sintomas psicológicos, como ansiedade e depressão, são os mais prevalentes nessa população.

**Objetivo:**

Determinar a associação entre ansiedade, depressão e qualidade de vida de cuidadores de pacientes com câncer em cuidados paliativos.

**Materiais e métodos:**

Foi utilizado um delineamento quantitativo, descritivo e correlacional. Participaram 190 cuidadores primários recrutados de hospitais colombianos e programas de assistência domiciliar. Foram utilizados o questionário Quality of Life in Life-Threatening Illness-Family Carer (QOLLTI-FT), o Beck Depression Inventory-II (BDI-II) e o Beck Anxiety Inventory (BAI). A análise foi realizada com o SPSS Statistics 26.0, utilizando estatística descritiva e inferencial.

**Resultados:**

O sexo predominante foi o feminino e o nível de escolaridade foi o ensino médio. A média de idade dos cuidadores foi de 44,15 anos e a média de idade dos pacientes foi de 64,51 anos. O tempo médio como cuidador foi de 33,79 ± 64,77 meses. Os resultados mostram correlações entre ansiedade e status de cuidador (p≤0,000), ansiedade e uso de TIC (p≤0,006). Também houve correlações entre ansiedade, qualidade de vida e status de cuidador (p≤0,000) e entre depressão e qualidade de vida total (p≤0,001).

**Discussão:**

Também foram encontradas correlações entre depressão e qualidade de vida e entre horas de cuidado diário e o nível de dependência do paciente oncológico. Isso implica na necessidade de manejar os sintomas psicológicos assim que o familiar é diagnosticado para prevenir alterações em sua qualidade de vida que possam afetar seu bem-estar.

**Conclusões:**

Cuidadores com depressão moderada ou grave foram mais propensos a relatar sintomas de tristeza, sentimentos de punição, auto aversão, pensamentos ou desejos suicidas, indecisão, irritabilidade, alterações no apetite, dificuldade de concentração e cansaço ou fadiga. Entre cuidadores com depressão leve, perda de interesse em sexo, agitação e fracasso passado foram identificados. Estratégias para aconselhamento psicoemocional, educação e suporte para cuidadores são necessárias.

## Introduction

According to the Pan American Health Organization (PAHO) and the World Health Organization (WHO), cancer is the second leading cause of death in the Region of the Americas, with an estimated 4 million new cases and 1.4 million deaths in 2020^1^,^2^. The global burden is projected to increase to 30 million new cancer cases by 2040, with the greatest increase in low- and middle-income countries^1^,^2^. In Latin American countries such as Colombia, 113,221 new cases and 54,987 deaths were reported in 2020^3^. 

This situation has raised global alarm, especially in developing countries with barriers to progress and cost overruns for health systems. Sometimes, this situation is due to a lack of preparedness of systems to meet the needs of people with cancer and their caregivers, leading to late interventions that can complicate disease management^4^. 

Advances in cancer diagnosis, treatment, and palliative care have improved patient survival and shifted care from highly complex institutions to outpatient centers or patients’ homes. These changes have resulted in family caregivers assuming caregiving activities for the person with cancer^5^, experiencing changes in their daily roles, and feeling overburdened, which negatively affects their health and quality of life^6^. Several studies indicate the prevalence of mental disorders in cancer patients’ caregivers that interfere with their self-care^7^,^8^. Common feelings among caregivers include anxiety, distress, and depression^9^,^10^, as well as changes in roles at home, school, or work^11^. In referring to anxiety and depression, Watson and Clark^12^ reported a temperamental sensitivity to negative stimuli such as fear, anxiety, sadness, guilt, hostility, dissatisfaction, hopelessness, somatic complaints, and a negative self-image. 

Depression is often accompanied by changes in sleep, appetite, and psychomotor function, along with reduced attention, concentration, and decision-making ability. It may also involve a loss of self-confidence, feelings of inferiority, worthlessness or guilt, a sense of hopelessness, and recurrent thoughts of death, including suicidal ideation, planning, or attempts^13^. 

On the other hand, anxiety, as defined by Beck^14^, is an emotional and physiological response to a perceived threat or danger. It can manifest through symptoms such as tremors, fear, and palpitations, among others. Beck emphasizes that anxiety is not solely an emotional phenomenon but also has cognitive and somatic dimensions^14^. 

Regarding caregivers' quality of life, Cohen^15^ defines it as subjective well-being that reflects the gaps between an individual's hopes and expectations and their current experiences. This concept encompasses aspects such as caregiver status, patient well-being, quality of care, perspectives, environment, finances, and relationships. 

In most cases, cancer care focuses primarily on the patient’s needs and the instructions healthcare professionals provide to caregivers for home care. However, little to no specific attention is given to the caregivers despite the emotional and physical burden they bear. This study goes beyond the limited existing scientific evidence by focusing on the well-being of caregivers, who are fundamental in the cancer care process. By analyzing anxiety, depression, and quality of life among caregivers of palliative care patients with cancer, the study aims to advance knowledge in this area and identify specific needs that could inform future, more effective support interventions. The present study aims to determine the association between anxiety, depression, and quality of life of caregivers of palliative care patients with cancer. 

## Materials and Methods

**Study Design and Participants**

A descriptive correlational study was conducted between January and August 2023, involving 190 family caregivers of cancer patients who had no curative treatment options and received care from either inpatient or community-based palliative care teams. Family caregivers over 18 years old from four regions of the country, who were the primary caregivers responsible for patient care, were invited to participate in this study. Caregivers were excluded from the study if they had cognitive impairment, had participated in a previous study, had previously cared for another person with cancer, or were hired to provide care. The participants were selected using convenience sampling. 

** Measurements**

The Sociodemographic Characterization Form was used to collect information on sociodemographic characteristics, medical history, and variables related to the caregiver’s profile. 

 The Quality of Life in Life-Threatening Illness – Family Carer Version (QOLLTI-F) was used to assess the quality of life of the palliative care caregivers. The Latin American-Spanish version of the QOLLTI-F has 16 items and seven dimensions. The scale has response options on a scale of 0 to 10, where the higher the score, the higher the quality of life. Scores range from 0 to 160 points. The scale has an internal consistency with a Cronbach’s alpha of 0.82 for the Latin American-Spanish version^15^. 

The Beck Depression Inventory-II (BDI-II) was used to identify the presence of depression in the family caregivers. The BDI-II consists of 21 items that are self-rated by the participants by marking the statement that best fits their current situation. Total scores on the scale range from 0 to 63, with participants falling into one of four groups: 0-13, minimal depression; 14-19, mild depression; 20-28, moderate depression; and 29-63, severe depression. This inventory had excellent internal consistency with a Cronbach’s alpha of 0.91 in the Colombian population^13^. 

The Beck Anxiety Inventory (BAI) was used to assess somatic symptoms of anxiety in the family caregivers. This questionnaire consists of 21 questions on a response scale of 0 to 3, with a score range of 0 to 63 points. The cut-off points report minimal anxiety from 0 to 5 points, mild anxiety from 6 to 15 points, moderate anxiety from 16 to 30 points, and severe anxiety from 31 to 63 points. This scale has been validated in Spanish and shows a unidimensional structure and a Cronbach’s alpha of 0.94^13^. 

**Data collection**

Participants were recruited from third- and fourth-level hospitals and home care programs in four Colombian cities: Bogotá, Medellín, Valledupar, and Cali. Caregivers were invited to participate in the study in hospital rooms or via telephone contact for those at home. Research assistants were trained to administer the instruments, present the study objectives, review the inclusion criteria, and invite caregivers to participate. Those who agreed to participate signed the informed consent form. The research instruments were interviewer-administered in hospital rooms or during home visits. 

**Data Analysis**

Data were tabulated in a Microsoft Excel database and then analyzed using SPSS Statistics version 26. Descriptive statistics were used to describe the sociodemographic characteristics; frequencies, means, and standard deviations were calculated. Spearman’s correlation coefficient and Kruskal-Wallis and Pearson’s chi-square tests were employed to determine the relationship between participants’ sociodemographic characteristics and their relationship with anxiety, depression, and quality of life. The information is stored in the Mendeley dataset^16^. 

**Ethical Considerations**

The Research Ethics Committee of Universidad Antonio Nariño approved the research in the ordinary session of August 16, 2022, with code 2022211. According to Resolution 008430 of 1993 from the Colombian Ministry of Health, the research posed minimal risk to participants^17^. Bioethical principles were upheld, informed consent was obtained, participants' autonomy was respected, and they were free to withdraw voluntarily. 

## Results

**Caregivers’ Sociodemographic characteristics**

A total of 190 caregivers participated in the study, and their sociodemographic characteristics are shown in Table 1. 

**Table 1:** Characteristics of Caregivers of Palliative Care Patients. Colombia 2023. n=190

Characteristic	n(%)
Caregiver conditions and sociodemographic profile	
Do you have any medical diagnoses?	
No	143 (75.30)
Yes	47 (24.70)
Sex	
Female	147 (77.40)
Male	43 (22.60)
Age	
< 30 years	47 (24.70)
31 - 43 years	50 (26.30)
44 - 56 years	47 (24.70)
> 57 years	46 (24.20)
Level of education	
Elementary school	38 (20.00)
Bachelor’s degree	41 (21.60)
High school	65 (34.20)
Technician	46 (24.20)
Marital status	
Married	54 (28.40)
Single/Divorced	86 (45.30)
Cohabitation	50 (26.30)
Religion	
Have a religious practice	174 (91.60)
No religious practice	16 (8.40)
Occupation	
Employee	77 (40.50)
Homemaker	72 (37.90)
Self-employed	34 (17.90)
Retired	7 (3.70)
Socioeconomic stratification	
Lower-low	34 (17.90)
Low	80 (42.10)
Lower-middle	54 (28.40)
Middle	20 (10.50)
Upper-middle	2 (1.10)
Do you care for the person after having been diagnosed?	
No	33 (17.40)
Yes	157 (82.60)
Burden and support perception	
Sole caregiver	
No	113 (59.50)
Yes	77 (40.50)
Time as a caregiver (months)	
< 5	50 (26.30)
6-12	49 (25.80)
13-36	47 (24.70)
> 37	44 (23.20)
Number of hours of daily caregiving	
Up to 8 hours	49 (25.80)
8-12 hours	47 (24.70)
> 12 hours	94 (49.50)
Do you have previous experience as a caregiver?	
No	115 (60.50)
Yes	75 (39.50)
PULSES Profile	
Fully independent	120 (63.20)
Requires assistance	33 (17.40)
Fully dependent	37 (19.50)
The person you are caring for is a	
Friend	14 (7.40)
Spouse	45 (23.70)
Child	10 (5.30)
Parent, sibling	99 (52.10)
Other (grandparent, grandchild, cousin)	22 (11.60)
Media and Information	
Use of ICT for care?	
No	38 (20.00)
Yes	152 (80.00)
ICT Proficiency	
High	42 (22.10)
Low	38 (20.00)
Middle	105 (55.30)
None	5 (2.60)
ICT used for care	
Computer, internet	71 (37.40)
Phone, internet	60 (31.60)
Television	59 (31.10)

ICT-Information and Communication Technology

**Anxiety and depressive symptoms and their association with quality of life in family caregivers of palliative care patients with cancer**

Regarding anxiety symptoms, 22 caregivers were found to have moderate to severe anxiety symptoms (14 and 8, respectively). Feeling hot, fear of the worst happening, being terrified or afraid, nervousness, fear of losing control, and faint or lightheadedness were common symptoms among caregivers with moderate anxiety. Caregivers with severe anxiety reported physical symptoms such as feeling hot, wobbliness in legs, heart pounding/racing, dizziness, feeling of choking, indigestion, and hot/cold sweats. In addition, symptoms include fear of the worst happening, unsteadiness, being terrified or afraid, nervousness, shaky/unsteady, fear of losing control, and inability to relax. 

Regarding symptoms of depression, 34 caregivers were found to have mild depression, 19 had moderate depression, and 25 had severe depression. Pessimism, loss of pleasure, guilty feelings, self-criticalness, crying, loss of interest, worthlessness, loss of energy, and changes in sleeping patterns were symptoms present in all three classification groups of depression. In addition, caregivers with moderate or severe depression were more likely to report symptoms of sadness, punishment feelings, self-dislike, suicidal thoughts or wishes, indecisiveness, irritability, changes in appetite, concentration difficulty, and tiredness or fatigue. Among caregivers with mild depression, loss of interest in sex, agitation, and past failure were identified as common symptoms. 

In terms of quality of life, the mean scores showed acceptable results for overall QOL. Below-average scores were found for the patient condition dimension and borderline scores for the caregiver’s state dimension. The internal consistency for the total scale domains was 0.75 (Table 2). 

**Table 2:** Quality of Life in Caregivers of Palliative Care Patients Colombia 2023. n=190

Quality of Life	Mean	SD	95% CI	Cronbach’s Alpha
Environment	15.30	3.78	14.59 – 15.67	0.75
Patient condition	3.86	2.87	3.45 – 4.27	
Caregiver own state	36.95	10.47	35.45 – 38.45	
Caregiver outlook	24.57	5.25	23.82 – 25.33	
Quality of care	16.91	3.20	16.45 – 17.36	
Relationships	16.99	3.11	16.55 – 17.44	
QOLLTI-F Total	114.42	20.88	111.43 – 117.41	

In terms of the sociodemographic characteristics of caregivers, statistically significant differences were found between anxiety and level of ICT proficiency, with non-use of ICT being a risk factor. As for depression, the number of hours of daily care, the patient’s level of dependency, and the average level of ICT use were identified as risk factors, with these variables showing statistically significant differences. The level of education and not being a sole caregiver were identified as protective factors with statistically significant differences for depression (Table 3). 

**Table 3:** Sociodemographic characteristics, anxiety, and depression in caregivers of palliative care patients. Colombia 2023. n=190*

	Suggests anxiety n=22	Without anxiety n=168	Prevalence ratio (95% CI)	P	Suggests depression n=78	Without depression n=112	Prevalence ratio (95% CI)	P
Caregiver conditions and sociodemographic profile								
Sex				0.10				0.01
Female	20 (13.60)	127 (86.40)	2.92 (0.71 - 12.02)		67 (45.60)	80 (54.40)	1.78 (0.03 - 3.05)	
Male	2 (4.70)	41 (95.30)	1		11 (25.60)	32 (74.40)	1	
Age								
< 30 years	7 (14.00)	43 (86.00)	1.09 (0.39 - 3.02)	0.85	17 (34.00)	33 (66.00)	0.72 (0.44 - 1.11)	0.19
31 - 43 years	6 (12.80)	41 (87.20)	1 (0.34 - 2.87)	>0.99	23 (48.90)	24 (51.10)	1.04 (0.68 - 1.59)	0.83
44 - 56 years	3 (6.50)	43 (93.50)	0.51 (0.13 - 1.92)	0.31	16 (34.80)	30 (65.20)	0.74 (0.45 - 1.22)	0.23
> 57 years	6 (12.80)	41 (87.20)	1		22 (46.80)	25 (53.20)	1	
Level of education								
Elementary school	5 (13.20)	33 (86.80)	1.34 (0.39 - 6.45)	0.63	9 (23.70)	29 (76.30)	0.44 (0.23 - 0.83)	0.006
High school	5 (7.70)	60 (92.30)	0.78 (0.22 - 2.76)	0.71	27 (41.50)	38 (58.50)	0.77 (0.51 - 1.16)	0.22
Technician	8 (17.40)	38 (82.60)	1.78 (0.57 - 5.48)	0.30	20 (43.50)	26 (56.50)	0.81 (0.52 - 1.25)	0.34
Bachelor’s degree	4 (9.80)	37 (90.20)	1		22 (53.70)	19 (46.30)	1	
Socioeconomic stratification								
Lower-low	5 (14.70)	29 (85.30)	2.94 (0.36 - 23.42)	0.27	6 (17.60)	28 (82.40)	0.35 (0.07- 1.68)	0.26
Low	10 (12.50)	70 (87.50)	2.5 (0.339 - 18.4)	0.37	34 (42.50)	46 (57.50)	0.85 (0.20 - 3.47)	0.83
Lower-middle	4 (7.40)	50 (92.60)	1.48 (0.176 - 12.47)	0.71	25 (46.30)	29 (53.70)	0.70 (0.45 - 1.09)	0.16
Middle	1 (5.00)	19 (95.00)	1		12 (60.00)	8 (840.00)	0.77 (0.48 - 1.22)	0.29
Upper-middle	2 (100)	0 (0.00)	----		1 (50.00)	1 (50.00)	1	
The caregiver cares for the person after having been diagnosed				0.91				0.78
No	4 (12.10)	29 (87.90)	1.05 (0.38 - 2.92)		7 (21.20)	26 (78.80)	1.20 (0.28 - 5.02)	
Yes	18 (11.50)	139 (88.50)	1		71 (45.20)	86 (54.80)	1	
Burden and support perception								
Sole caregiver				0.07				0.010
No	17 (15.00)	96 (85.00)	2.31 (0.89 - 6.01)		56 (49.60)	57 (50.40)	0.46 (0.23 - 0.92)	
Yes	5 (6.50)	72 (93.50)	1		22 (28.60)	55 (71.40)	1	
Number of hours of daily caregiving								
Up to 8 hours	4 (8.20)	45 (91.80)	0.69 (0.23 - 2.07)	0.51	26 (53.10)	23 (46.90)	1.73 (1.16 - 2.58)	<0.005
8-12 hours	7 (14.90)	40 (85.10)	1.27 (0.52 - 3.07)	0.59	23 (48.90)	34 (51.10)	1.72 (1.15 - 2.56)	<0.005
>12 hours	11 (11.70)	83 (88.30)	1		29 (30.90)	65 (69.10)	1	
ICT Proficiency								
None	3 (60.00)	2 (40.00)	5.04 (1.69 - 15.00)	0.006	2 (40.00)	3 (60.00)	1.40 (0.43 - 4.53)	0.59
Low	2 (5.30)	36 (94.70)	0.44 (0.09 - 2.14)	0.29	13 (34.20)	25 (65.80)	1.19 (0.62 - 2.29)	0.58
Middle	12 (11.40)	93 (88.60)	0.96 (0.36 - 2.55)	0.93	51 (48.60)	54 (51.40)	1.70 (1.01 - 2.85)	0.02
High	5 (11.90)	37 (88.10)	1		12 (28.60)	30 (71.40)	1	
PULSES Profile								
Requires human assistance	2 (6.10)	31 (93.90)	0.45 (0.11 - 1.87)	0.25	6 (18.20)	27 (81.80)	0.57 (0.26 -1.24)	0.12
Fully dependent	4 (10.80)	33 (89.20)	0.81 (0.28 - 2.27)	0.68	34 (91.90)	3 (8.10)	2.90 (2.19 - 3.83)	<0.001
Fully independent	16 (13.30)	104 (86.70)	1		38 (31.70)	82 (68.30)	1	

* Pearson’s chi-square test

The analysis against manifestations of anxiety and quality of life yielded statistically significant differences among caregivers in the dimension "caregiver status," with no other important results. On the contrary, statistically significant differences were found between the levels of depression and most of the caregivers' quality of life dimensions except for the patient's condition. Higher scores for the manifestation of moderate or severe depression were identified for the dimensions of environment, quality of care, and relationship (Tables 4 and 5). 

**Table 4:** Quality of life and anxiety in caregivers of palliative care patients. Colombia 2023. n=190

	Very low anxiety n=168		Moderate anxiety n=14		Severe anxiety n=8		p-value
	Median	Min-max	Median	Min-max	Median	Min-max	
Environment	15.00	1-20	15.00	7-20	15.00	5-20	0.68
Patient's condition	3.00	0-10	4.50	0-10	1.50	0-7	0.49
Caregiver status	38.00	19-60	37.00	15-45	27.50	0-40	0.00
Caregivers' perspective	27.00	11-30	27.00	13-30	22.00	15-28	0.30
Quality of care	18.00	2-20	18.50	10-20	15.50	9-20	0.26
Relationships	18.00	1-20	18.00	5-20	18.00	10-19	0.78
QOLLTI- total	118.00	68-160	113.50	68-133	100.00	49-120	0.06

* Kruskal-Wallis test

**Table 5:** Quality of Life in Caregivers of Palliative Care Patients Colombia 2023. n=190

	Minimal depression n=112		Mild depression n=34		Moderate depression n=19		Severe depression n=25		p-value
	Median	Min-max	Median	Min-max	Median	Min-max	Median	Min-max	
Environment	17.00	2-20	15.00	1-20	14.00	8-20	13.00	5-19	<0.001
Patient's condition	3.00	0-10	4.50	0-10	3.00	0-9	3.00	0-4	0.09
Caregiver status	41.00	19-60	34.50	22-55	31.00	15-46	29.00	0-42	<0.001
Caregivers' perspective	28.00	13-30	27.00	13-30	20.00	14-30	16.00	11-25	<0.001
Quality of care	19.00	2-70	18.00	9-20	15.00	8-20	13.00	12-18	<0.001
Relationships	18.00	1-20	17.00	5-20	17.00	10-20	16.00	10-19	0.010
QOLLTI- total	121.00	68-160	114.50	68-145	96.00	84-132	88.00	49-109	<0.001

* Kruskal-Wallis test

## Discussion

 Family caregivers of people with cancer in the study are mostly women who are caring for their parent, sibling, or spouse with cancer. They are working and responsible for caring for older adults, with limitations in developing self-care activities, similar to what has been reported in other studies^8^. The majority of this population belongs to the low and lower-middle socioeconomic strata, which limits their ability to care for themselves and their families due to the limited availability of resources. As in other studies, the caregivers have no experience or training in caregiving, are required to provide care for 12 hours per day, and most (although not all) have provided care for between 13 and more than 37 months, similar to what was reported in Ahmad Zubaidi et al.’s review^18^.

 It should be noted that although 31.9% of caregivers have a defined religious practice and 77.4% are satisfied with this dimension, there is a significant percentage of dissatisfaction, which is striking when we consider that religious practice and the exercise of spirituality are considered factors that help to overcome negative aspects corresponding to the psychological dimension. This situation was also found in the study by Asano et al.^19^, where negative religious coping was associated with greater depressive symptoms, and many of the caregivers who experienced depression had it mitigated by positive religious coping. Similarly, other supports identified by caregivers, such as healthcare professionals, family members, and social institutions, are perceived as insufficient to meet the needs of caregivers of people with cancer.

 Concerning family support, Rojas-García et al^20^. say in their study that families who have an adult relative with advanced cancer in palliative care face challenges that begin with accepting the diagnosis, changing family routines, and coping with the disease and circumstances that create different types of needs. This situation means that in addition to the caregiver, other family members are experiencing significant changes that may affect how they support the cancer patient’s caregiver.

 In terms of anxiety, although scores are reported to be low, 22 caregivers have moderate or severe anxiety; some may be related to uncertainty about the future, as evidenced by anxiety, nervousness, and fear of losing control. Similarly, regarding depression scores, the majority of caregivers have mild depression; however, 44 caregivers have moderate or severe depression. The most prominent symptoms are related to sadness, pessimism about the future, loss of pleasure, guilty feelings, lack of self-confidence, self-criticalness, crying, lack of interest, indecisiveness, loss of energy, sleep problems, irritability, lack of appetite, tiredness and fatigue, and loss of interest in sex. Chakraborty et al.^21^ and Fauziah et al.^22^ found similar symptoms in caregivers participating in their respective studies.

 These symptoms are related to the caregiver burden, which Guerrero-Gaviria et al.^23^ defined as a set of physical, psychological, and socioeconomic problems caused by the caregiving role that alters multiple aspects of daily life, such as interpersonal relationships, caregiving skills, emotional balance, and personal aspirations. In the case of the family caregivers in this study, changes in interpersonal relationships (as evidenced by lack of satisfaction with support received from others), changes in emotional balance, and personal aspirations (as evidenced by uncertainty about the future and perceptions of themselves and their caregiving) are evidence of caregiver strain. Lee et al.’s study^24^ shows a positive correlation between unmet needs and the burden of care. Health and psychological problems, family and social support, information, religious and spiritual needs, and practical support needs are some of the subdomains of unmet needs significantly correlated with the burden of care^24^.

 Bivariate and multivariate analyses confirm how anxiety is associated with the caregiver’s state and how caregiver depression can affect all dimensions of the caregiver’s quality of life. Similar results were reported in the study by Washington et al.^25^, which described the comfort needs of family caregivers of outpatient cancer patients, such as understanding, self-efficacy, meaning-making, informal support, formal support, resources, and self-care, all of which correspond to five out of the eight domains of care identified by the National Consensus Project for Quality Palliative Care’s Clinical Practice Guidelines. Along the same line, the review by Guerra-Martín et al.^10^ found that the more extended care was needed over time, the greater the tension and emotional burden of caregivers, resulting in poorer quality of life. 

 It is also important to mention how non-use of ICT by caregivers may be related to anxiety, something relevant considering that much of the information about caring for a family member with cancer is obtained primarily through the Internet or by calling peer caregivers or healthcare professionals. Likewise, it is also important to mention how the number of hours of care, the level of dependency, and the level of ICT proficiency become risk factors for the occurrence of depression. In this regard, studies such as Tay et al. highlight how the number of caregiving tasks and caregiver burden were associated with caregiver self-care behaviors.^8^ Studies such as Li et al.^26^ and Darley et al.^27^ show how e-health interventions are a convenient way to support informal caregivers of cancer patients and can mitigate depression and improve the quality of life for informal caregivers. However, it is important to note that these interventions did not significantly reduce the caregiver burden^26^,^27^.

 All of the above leads us to think about the need to work on a dyadic cancer care model involving the cancer patient and the family caregiver. These cancer patient-caregiver dyads should be approached in an interdisciplinary manner so that patients and caregivers receive the necessary guidance, support, and training to address both cancer progress and instrumental care at home, as well as those related to the caregiver’s well-being and self-care. To the extent that caregivers are cared for, and their quality of life and well-being are ensured during this experience over time, there will be better outcomes in the quality of care received by cancer patients. Molassiotis and Wang^28^ agree on the need to develop psychoeducation, skills training, and therapeutic counseling interventions as they have the potential to help reduce the burden of care and address information needs and coping strategies. Health literacy is also being reintroduced to refer to the caregivers’ skills and competencies needed to use health information to make informed decisions^28^.

## Conclusions

The characteristics of family caregivers of people with cancer align with those reported in previous studies: the majority are middle-aged women who provide over 12 hours of care daily to their partner or parent, often for more than a year. While most caregivers exhibited mild levels of depression and anxiety, some experienced moderate to severe symptoms, potentially leading to caregiver overburden and negatively affecting their quality of life. Protective factors identified in this study include a high level of education, average use of ICT, and sharing caregiving responsibilities, which nursing and interdisciplinary teams should consider. These findings highlight the need for targeted nursing interventions that not only address the patient’s needs but also actively support caregivers, thereby improving their quality of life. Enhancing the quality of nursing care for both patients and families in palliative cancer care could be achieved by integrating strategies that reduce caregiver burden and promote mental well-being.
